# Quantitative optical coherence tomography angiography of macular vascular structure and foveal avascular zone in glaucoma

**DOI:** 10.1371/journal.pone.0184948

**Published:** 2017-09-21

**Authors:** Jaewan Choi, Junki Kwon, Joong Won Shin, Jiyun Lee, Saem Lee, Michael S. Kook

**Affiliations:** 1 Central Seoul Eye Center, Seoul, Republic of Korea; 2 Department of Ophthalmology, University of Ulsan, College of Medicine, Asan Medical Center, Seoul, Republic of Korea; Bascom Palmer Eye Institute, UNITED STATES

## Abstract

**Objective:**

The study aimed to evaluate the quantitative characteristics of the macular vessel density (VD) and foveal avascular zone (FAZ) in glaucoma using optical coherence tomography angiography (OCT-A).

**Design:**

Cross-sectional, age- and sex-matched case–control study.

**Methods:**

Fifty-two eyes of 52 patients with primary open angle glaucoma and 52 eyes from 52 healthy participants were recruited retrospectively. OCT-A was performed on a 3 x 3-mm macular region centered on the fovea. OCT-A scans were manually graded to define the FAZ. Parafoveal VD in superficial and deep retina were analyzed in the circular- and quadrant-segmented zone. The FAZ parameters included size, perimeter, and circularity index. The regression analysis among VD and FAZ-related parameters and ocular parameters was performed, and the diagnostic ability was calculated with refractive error adjusted.

**Results:**

For both groups, the mean age and the sex ratio was not different between groups. With refractive error adjusted, the average macular VD was lower in glaucoma than in the control group for superficial (P = 0.013), deep (P<0.001), and the whole retina (P = 0.002). There were increased FAZ perimeter and decreased FAZ circularity index in glaucoma when compared with controls (P<0.001). In the multivariate regression models, FAZ circularity index were significantly associated with decreased peripapillary RNFL thickness (P = 0.007) and macular GCIPL thickness (P = 0.009) measured by OCT. The refractive-error adjusted area under receiver operating characteristics was highest for FAZ circularity index (0.905; 95% CI, 0.844–0.966), followed by temporal deep retinal VD (0.870; 95% CI, 0.803–0.937) and FAZ perimeter (0.858; 95% CI, 0.784–0.932).

**Conclusions:**

Decreased macular VD, increased FAZ perimeter, and decreased FAZ circularity index were observed in eyes with glaucoma using OCT-A. With refractive error adjusted, these parameters showed considerable diagnostic value for glaucoma. FAZ circularity index may be a novel biomarker representing disruption of the parafoveal capillary network in glaucoma, as supported by its association with structural parameters.

## Introduction

Hypotheses associated with the development of primary open angle glaucoma (POAG) and normal tension glaucoma (NTG) can be largely classified into those involving vascular factors,[1–5] and those involving biomechanical stress such as intraocular pressure (IOP) or thin lamina cribrosa.[6–9] Although IOP has been regarded as the most important risk factor in the management of glaucoma, reducing IOP does not always guarantee the cessation of the disease progression. Recent studies have proposed that POAG and NTG may represent a continuum of open-angle glaucoma (OAG) that differs primarily in predominant causative risk factors, with higher IOP being important in POAG, whereas additional IOP-independent factors such as nocturnal hypotension or circadian fluctuation of mean ocular perfusion pressure may be more important in NTG.[1, 2, 10–12]

Various modalities have been developed and applied to assess the ocular blood flow (OBF), including color Doppler imaging, laser Doppler flowmetry, retinal vessel analyzer, laser speckle flowgraphy, and fluorescein angiography (FAG).[13–18] However, there is no consensus on measuring OBF, as modern hemodynamic assessment techniques provide only a partial description of the ocular circulation.[15] It may also be contributed to by individual differences in flow parameter readings in some OBF-measuring devices in clinical practice.[19]

Recently, a new non-invasive microvasculature imaging technique called optical coherence tomography angiography (OCT-A) was developed for visualization of both perfused vascular network and vascular abnormalities without the need of contrast eye.[20] Studies found out that vascular densities (VD) of the optic nerve head (ONH), peripapillary area, and macula have significant associations with structural glaucomatous damage expressed in the neuroretinal rim area, retinal nerve fiber layer (RNFL) thickness, and ganglion cell complex (GCC) thickness measured by optical coherence tomography (OCT).[21–23] A structure-to-function relationship also has been established between VD measured at different locations by OCT-A and visual field (VF) parameters in glaucoma.[22, 24, 25] For differentiating between glaucoma and healthy eyes, peripapillary VD had diagnostic accuracy comparable to the RNFL thickness measurement.[26]

In contrast, macular VD had significantly lower diagnostic abilities in OAG than did peripapillary VD.[27] We believe that the foveal avascular zone (FAZ) should be the primary focus in the analysis of macular vascular structure for demonstrating glaucoma pathogenesis. The degeneration or atrophic changes of macular capillaries may affect the shape and size of FAZ, which have been shown to be of both diagnostic and prognostic value in retinal diseases such as retinal vein occlusion and diabetic retinopathy.[28–33] However, the clinical meaning of FAZ in glaucoma is not well understood. To the best of our knowledge, no attempt to the present time has been made to investigate the disruption of the parafoveal capillary network in eyes with glaucoma.

The purpose of this study was to present a method to assess the VD of the macular area and the parafoveal capillary network quantitatively, to investigate the associations between these parameters and glaucomatous structural and functional damage, and to test its diagnostic ability for discriminating glaucoma from healthy eyes.

## Materials and methods

### Study subjects

The study conformed to the tenets of the Declaration of Helsinki. There was no need for patient consent as the data were analyzed retrospectively and anonymously. Asan Medical Center IRB Approval number is S2016-1987-0001. This was a retrospective, age- and sex-matched case–control study performed from April 1, 2016 to August 31, 2016 at the Central Seoul Eye Center and the Asan Medical Center after approval of the Institutional Review Board. The study conformed to the tenets of the Declaration of Helsinki.

We retrospectively recruited 52 eyes of 52 patients with OAG, and 52 eyes of 52 age- and sex-matched healthy control subjects. Inclusion criteria for both groups were (1) best-corrected visual acuity of 20/30 or better, refractive error between -12 and +3 diopter (D) spherical equivalent, (2) cylinder correction within 3 D, and (3) clear ocular media to prevent poor-quality imaging of the optic disc and macula.

OAG was defined as the presence of glaucomatous optic nerve appearance, associated structural and functional VF defect identified by RNFL photography and standard automated perimetry (Humphrey Field Analyzer II 850; 24–2 Swedish interactive threshold algorithm standard; Carl-Zeiss Meditec, Dublin, CA), and open angle on gonioscopic examination. A glaucomatous VF change was defined as (1) outside normal limit on glaucoma hemifield test or (2) three abnormal points with *P* less than 5% probability of being normal or one abnormal point with *P* less than 1% by pattern deviation, or (3) pattern standard deviation (PSD) of 5% if the VF was otherwise normal, confirmed by two consecutive tests. A VF measurement was considered to be reliable when false-positive/negative results were less than 15% and fixation losses were less than 20%. For each glaucoma patient, an age- and sex-matched healthy subject who visited the clinic during the same recruitment period was enrolled and served as a control subject. Healthy subjects had (1) IOP less than 21 mm Hg with no history of elevated IOP; (2) normal appearance of optic disc, intact neuroretinal rim, and RNFL; and (3) normal visual fields, defined as a PSD within 95% confidence limits and a glaucoma hemifield test result within normal limits. Exclusion criteria for both groups were (1) severe myopic disc and fundus changes impairing adequate ONH/ VF evaluation for glaucoma, (2) coexisting retinal or neurologic diseases that could affect the VF, (3) poor quality OCT-A or spectral domain OCT scans that had significant artifacts (poor signal strength < 8, loss of fixation, asymmetric illumination, or motion artifacts such as vessel doubling or edge duplication), and (4) a history of diabetes mellitus, retinal vaso-occlusive diseases such as retinal vein occlusion that can affect FAZ microvasculature, or prior eye surgery/ laser treatment. Patients with a history of uncomplicated cataract surgeries or systemic hypertension were not excluded. The affected eye was selected in patients with unilateral glaucoma, and if both eyes of a patient had glaucoma and met the inclusion criteria, one eye was randomly selected for entry. A healthy control eye was randomly selected in patients with no sign of glaucoma in both eyes.

Each subject underwent a comprehensive ophthalmic examination including visual acuity assessment, manifest refraction, slit-lamp biomicroscopy, gonioscopy, Goldman applanation tonometry, and dilated stereoscopic examination of the optic disc and retina. Subjects also underwent standard automated perimetry, spectral domain OCT peripapillary RNFL thickness measurement, macular ganglion cell and inner plexiform layer (GCIPL) thickness measurement, and OCT-A (Cirrus 5000HD equipped with ZEISS AngioPlex^™^; Carl-Zeiss Meditec, Dublin, CA) examination. The intereye symmetry, which describes the correlation in retardation measurements between the eyes of a subject, was not used for analysis because only one eye from each of the glaucoma and control subjects was used.

### OCT-A image acquisition

All eyes were scanned using an OCT-A system offering optical microangiography algorithm as described earlier. OCT-A volume scans (3 x 3 mm) centered on the fovea were separately acquired. The built-in segmentation algorithm automatically detects the boundaries of the retinal layers from the structural OCT cross-sectional images. A trained OCT-A user (S.L.) acquired the images, and the automatically segmented superficial and deep retinal capillary plexus were individually reviewed by one of the investigators (J.C.) for quality evaluation, and unqualified scans were excluded.

### Assessment of macular vessel density

Assessment of macular VD was performed using an image processing algorithm written in MATLAB software (The MathWorks, Inc., Natick, MA). Each 3 x 3 mm macular superficial and deep retinal vessel image was analyzed. Briefly, the vessel edges in a certain image are first accentuated by using a low-pass filter and the resultant image subtracted from the original image. The result is then thresholded for intensity and object size to isolate vessels and remove noise. A median filter to smooth vessel edges is then applied to the binary image. The VD was expressed as a percentage by taking the ratio of the total vessel area (all pixels with a ratio value between 0.7 and 1.0) to the total area of region of interest (size of the image in pixels). VD was separately evaluated at superficial and deep retinal layers.

**Fig 1:**
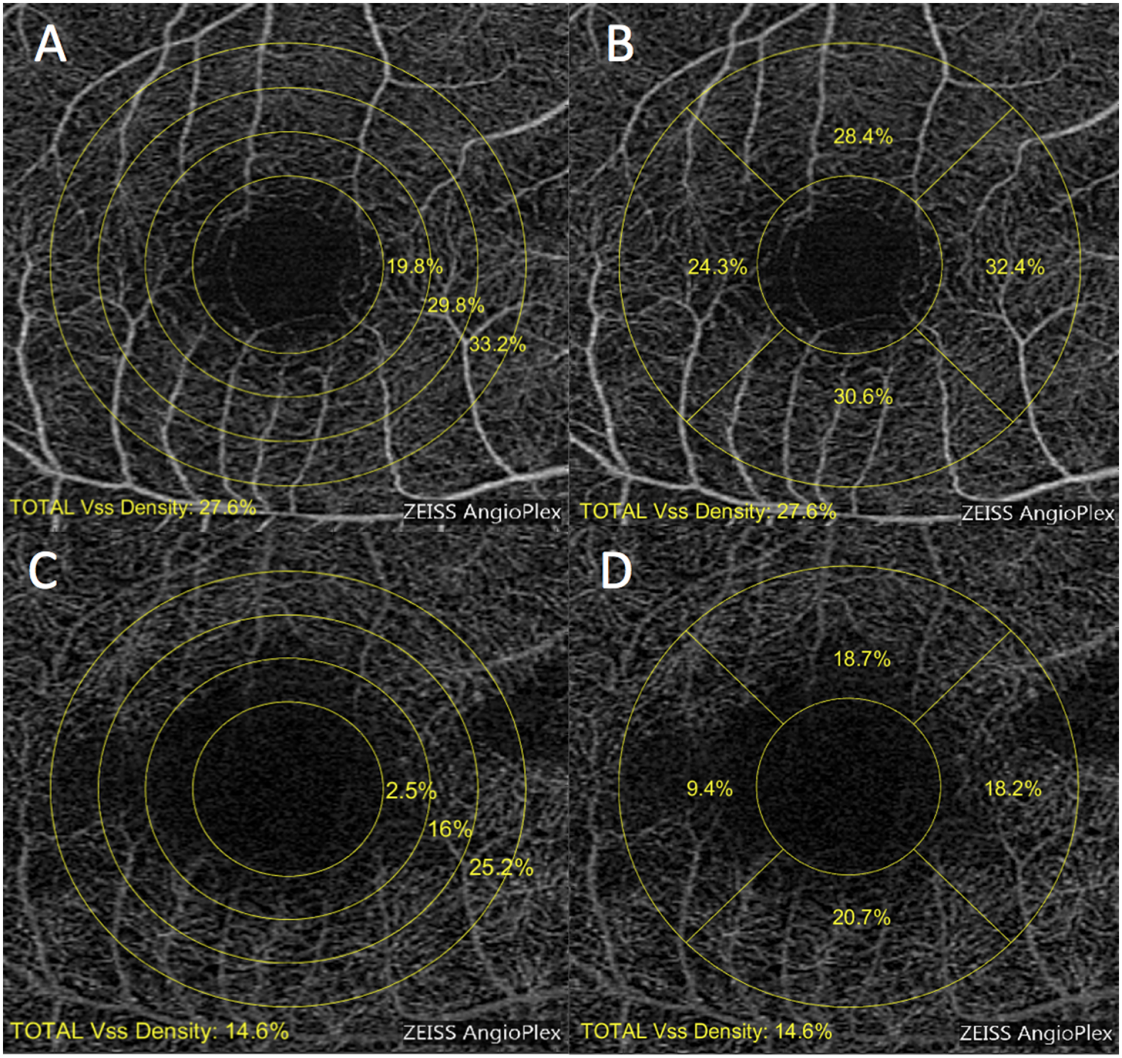
Macular vascular density at the different levels of 3 x 3-mm retinal vascular plexus. (A) Superficial retinal vascular plexus in circular segmentation. (B) Superficial retinal vascular plexus in quadrant segmentation. (C) Deep retinal vascular plexus in circular segmentation. (D) Deep retinal vascular plexus in quadrant segmentation. For the circular segmentation, vascular density was calculated in three circular regions of different radius after excluding the adjacent inner circle area. Each region corresponding to C1, C2, and C3 had a 1.5-mm, 2-mm, and 2.5-mm radius from the center. The central area of 1-mm radius was excluded, most of which included the FAZ area. Similarly, quadrant segmentation was performed in four sectors, namely, temporal (T), superior (S), nasal (N), and inferior (I) in the region of 1-mm to 2.5-mm radius.

Two sorts of regional segmentation were performed for superficial and deep retinal vessel images. For the circular segmentation, VD was measured at various distances from fovea: three concentric circular regions from 1.0 to 2.5 mm radius with 0.5-mm interval (Fig 1A and 1C; C1, 1.0–1.5 mm; C2, 1.5–2.0 mm; C3, 2.0–2.5 mm). For the quadrant segmentation, VD was measured in the parafoveal sectors after excluding the fovea-centered 1.0-mm radius area, namely, temporal, superior, nasal, and inferior quadrant inside the circular 2.5-mm radius area (Fig 1B and 1D).

### Assessment of foveal avascular zone

We analyzed FAZ with respect to size, perimeter, and circularity index. OCT-A scans of the superficial and deep retinal vasculature were exported and independently assessed by two trained graders (J.W.S. and J.K.) using ImageJ (version 1.49, National Institutes of Health, Bethesda, MD, USA). The image scale was set using known image size of 452 x 452 pixels. Because each scan length is 3 mm horizontally and vertically, a pixel aspect ratio of 1.0 was set, resulting in a scale of 150.67 pixels per mm. The whole edge points were manually connected to each other along the borderline of the identifiable capillary network in the parafoveal area. FAZ size and perimeter were calculated by the software from the comparison of the 9 mm^2^ total image size.

Circularity index is a measure of compactness of a shape relative to a circle. The circularity index of a circle is 1.0. Thus, a ratio closer to 0 indicates an irregular shape, and that closer to 1.0 indicates a circular shape. The circularity index is calculated as a function of the perimeter and the area of a shape.

$$Circularity\;index=\frac{4\pi \times area}{perimeter^{2}}$$

Our hypothesis for integrating FAZ circularity index into the analysis is that if disruption of parafoveal capillary network progresses, FAZ shape would be less likely that of a perfect circular shape, which may lead to a decrease in FAZ circularity index. Circularity index has been applied in other fields of ophthalmology to describe the perfectness of capsulorhexis in cataract surgery or the prognosis of geographic atrophy.[34, 35]

### Statistical analysis

Descriptive statistics for baseline demographics and clinical parameters associated with functional and structural glaucomatous damage were calculated as the mean deviation (MD) and PSD for normally distributed variables. Functional parameters included MD, PSD, and visual field index (VFI) measured by Humphrey VF analyzer, and structural parameters included clock-hour, quadrant, average macular GCIPL thickness, and average peripapillary RNFL thickness measured by OCT. Categorical variables were compared using the chi-square test. Differences in continuous variables between groups were determined using independent sample t-tests. For the assessment of VD and FAZ-related parameters, the superficial and deep retinal VD in each circular and quadrant segmentation were compared between glaucoma and the control group. To adjust for the refractive error, analysis of covariance (ANCOVA) was also performed with spherical equivalent (SE) set as a covariate. Associations between macular VDs, FAZ-related parameters, and structural and functional glaucomatous damage were determined using linear regression analysis. To assess the reliability of the measurements of FAZ size and circularity index, interclass correlation coefficients (ICCs) with 95% confidence intervals were calculated for intersession and intergrader (J.K. and J.W.S.) measurements for glaucoma subjects (n = 30), who were randomly selected and scanned on two separate occasions.

To investigate the diagnostic ability of VD and FAZ-related parameters, area under the receiver operating characteristics (AUROC) curves were calculated both before and after refractive error adjustment. Pairwise comparison of refractive error-adjusted ROC curves was performed to test the diagnostic ability of macular VDs and FAZ-related parameters using the method of DeLong et al. using the software (MedCalc v. 17; MedCalc Statistical Software, Marakierke, Belgium).[36] For multiple comparisons, the Bonferroni correction was used to adjust for type I error.

## Results

### Study population

Table 1 presents the patient demographics and biometric parameters, including refractive error, IOP, and VF, peripapillary RNFL thickness, and macular GCIPL thickness parameters for each group. Age and sex ratio and IOP did not differ between glaucomatous and healthy eyes. Refractive error was more myopic in the glaucomatous eyes than in the healthy control group (*P*<0.001). There were statistically significant differences between glaucomatous and healthy eyes in all VF parameters and OCT parameters except for 3 o’clock and 4 o’clock RNFL thickness between the groups (*P*<0.001).

**Table 1:** Clinical characteristics of the study patients.

	Normal controls (n = 52)	Glaucoma (n = 52)	P Value
Demographic and clinical parameters			
Age (years)	52.13 ± 14.79	52.06 ± 14.45	0.98
*- 29 (n)*	*5*	*5*	
*30–39 (n)*	*5*	*5*	
*40–49 (n)*	*12*	*12*	
*50–59 (n)*	*14*	*14*	
*60–69 (n)*	*9*	*9*	
*70–(n)*	*7*	*7*	
Sex (male: female)	33:19	33:19	
Spherical equivalent (D)	-0.53 ± 2.06	-3.35 ± 3.30	<0.001*
IOP (mmHg)	14.1 ± 3.01	14.2 ± 4.34	0.76
Visual field parameters			
MD (dB)	-0.57 ± 1.80	-8.74 ± 7.29	<0.001*
PSD (dB)	1.83 ± 0.48	9.11 ± 4.31	<0.001*
VFI (%)	98.52 ± 1.43	75.74 ± 20.04	<0.001*
Peripapillary RNFL thickness			
Clock-hour measurement			
1	104.56 ± 18.10	80.56 ± 19.92	<0.001*
2	84.29 ± 17.30	71.17 ± 13.21	<0.001*
3	62.41 ± 10.59	61.13 ± 12.60	0.585
4	63.96 ± 11.22	60.89 ± 12.90	0.208
5	97.21 ± 19.47	71.96 ± 15.62	<0.001*
6	134.73 ± 26.92	72.53 ± 26.27	<0.001*
7	146.69 ± 21.59	70.86 ± 28.00	<0.001*
8	77.46 ± 15.82	55.06 ± 15.52	<0.001*
9	58.56 ± 9.19	51.84 ± 12.10	0.002*
10	88.83 ± 16.59	67.06 ± 19.44	<0.001*
11	136.21 ± 21.25	91.04 ± 28.26	<0.001*
12	115.71 ± 23.36	80.85 ± 25.68	<0.001*
Quadrant measurement			
Superior	118.79 ± 14.16	84.10 ± 20.88	<0.001*
Inferior	126.21 ± 17.59	71.73 ± 20.36	<0.001*
Temporal	74.04 ± 12.19	58.00 ± 13.52	<0.001*
Nasal	71.06 ± 11.58	64.42 ± 11.07	0.004*
Average	97.54 ± 9.42	69.60 ± 12.98	<0.001*
Macular GCIPL thickness			
Average	83.95 ± 5.08	64.94 ± 10.09	<0.001*

IOP = intraocular pressure; MD = mean deviation; PSD = pattern standard deviation; VFI = visual field index; OCT = optical coherence tomography; RNFL = retinal nerve fiber layer, GCPIL = gangion cell and inner plexiform layer.

*Statistical significance was found using independent samples t-test (P<0.05).

### Quantitative assessment of macular VD and FAZ

**Fig 2:**
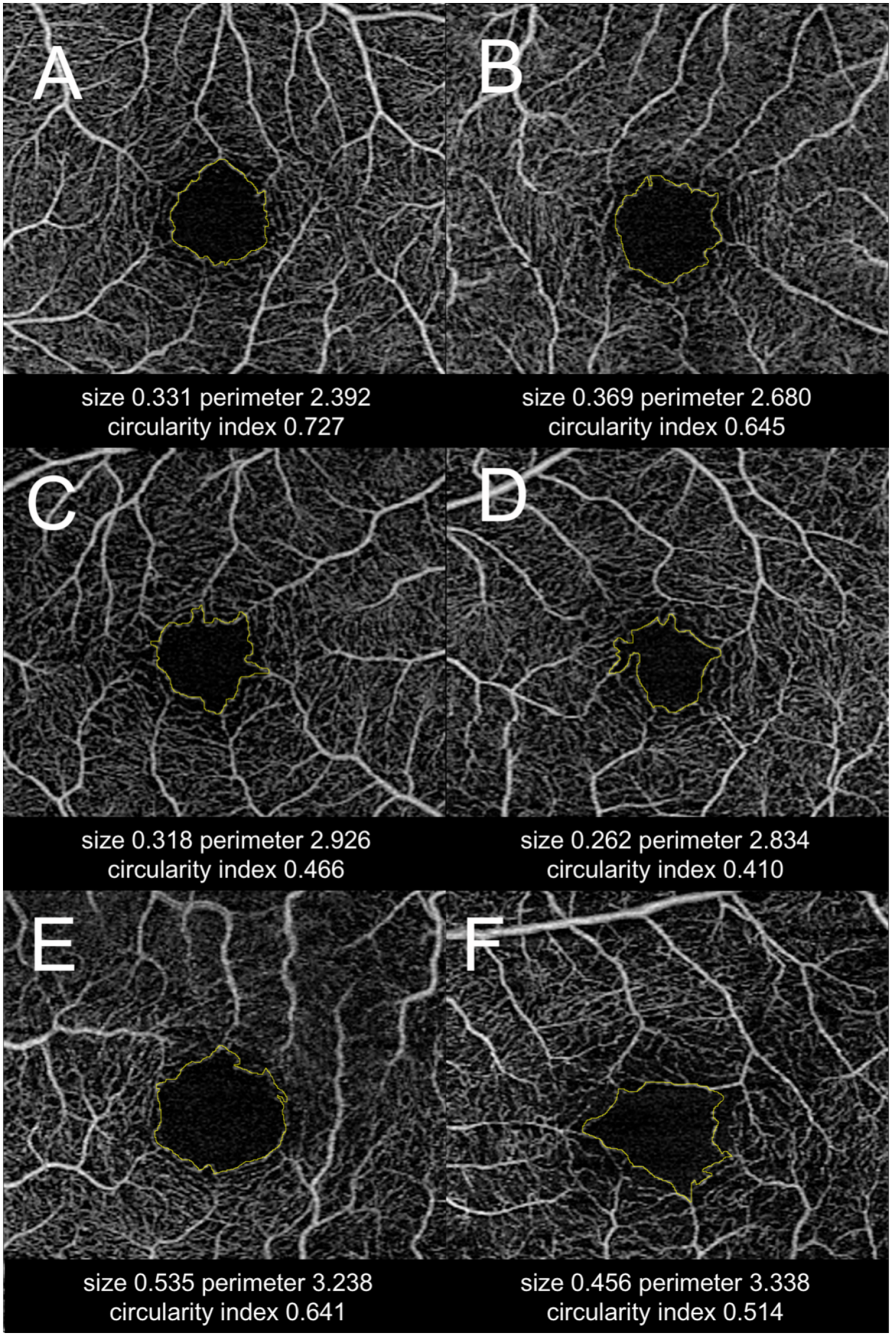
Representative cases with different foveal avascular zone (FAZ) assessment. Two healthy cases (A, B) and four glaucoma cases (C, D, E, F). (A, B) Healthy eyes have a relatively smaller FAZ size and a higher circularity index. (C, D) Glaucoma patients with remarkable foveal capillary dropout, which is reflected in lower circularity index. (E, F) Advanced glaucoma patients with notably enlarged FAZ size (E) or distorted FAZ shape (F).

The comparisons of macular VD and FAZ-related parameters assessed by OCT-A are presented in Table 2. After adjustment for refractive error, the macular VDs were significantly lower in most quadrant- and circular-segmented areas except for a few superficial areas (superior and nasal quadrant zone, and C1 circular zone) in the eyes with OAG. Macular VD was higher for the superficial vascular plexus, when compared with deep vascular plexus at all the analyzed zones in both glaucomatous and control group (P<0.05, paired t-test). On the FAZ-related parameter analysis, it is notable that FAZ size was not different between groups, but there was a higher FAZ perimeter and lower FAZ circularity index in the eyes with POAG regardless of the adjustment of refractive error (P<0.001). Representative cases are shown in Fig 2.

**Table 2:** Assessment of macular vascular density and foveal avascular zone by OCT angiography.

	Normal controls (n = 52)	Glaucoma (n = 52)	P value	P value adjusted for refractive error (SE)†
**Macular vascular density (%)**				
Quadrant zone				
Temporal *superficial*	30.78 ± 7.41	26.91 ± 7.08	0.008*	0.005*
*deep*	23.99 ± 6.50	16.53 ± 6.77	<0.001*	<0.001*
*whole retina*	39.00 ± 8.01	32.86 ± 8.03	<0.001*	<0.001*
Superior *superficial*	30.41 ± 8.09	28.26 ± 7.71	0.17	0.054
*deep*	26.20 ± 7.89	22.09 ± 8.39	0.011*	0.005*
*whole retina*	39.16 ± 9.30	35.68 ± 9.32	0.06	0.022*
Inferior *superficial*	29.53 ± 7.05	25.44 ± 6.64	0.003*	0.006*
*deep*	25.96 ± 7.27	20.81 ± 7.29	<0.001*	<0.001*
*whole retina*	38.26 ± 8.16	32.59 ± 8.11	0.001*	0.001*
Nasal *superficial*	30.08 ± 7.88	27.63 ± 8.60	0.13	0.073
*deep*	24.44 ± 7.25	22.09 ± 8.39	0.011*	0.009*
*whole retina*	38.17 ± 8.85	34.56 ± 10.22	0.057	0.035*
Circular zone				
C1 *superficial*	25.87 ± 7.81	23.97 ± 7.23	0.20	0.10
*deep*	14.48 ± 6.37	10.83 ± 6.51	0.005*	0.003*
whole retina	31.18 ± 8.38	28.34 ± 8.16	0.084	0.040*
C2 *superficial*	30.57 ± 6.79	27.59 ± 6.56	0.025*	0.012*
*deep*	26.98 ± 6.88	20.71 ± 7.79	<0.001*	<0.001*
*whole retina*	39.62 ± 7.63	34.70 ± 7.90	0.002	0.001*
C3 *superficial*	32.29 ± 6.59	28.35 ± 6.79	0.003*	0.002*
*deep*	29.60 ± 6.97	24.31 ± 7.48	<0.001*	<0.001*
whole retina	42.01 ± 7.68	36.39 ± 8.23	<0.001*	0.001*
Overall				
*superficial*	30.08 ± 6.83	27.06 ± 6.70	0.025*	0.013*
*deep*	25.15 ± 6.40	19.91 ± 7.14	<0.001*	<0.001*
*whole retina*	38.64 ± 7.66	33.93 ± 7.98	0.003*	0.002*
**Foveal avascular zone**				
FAZ size (mm^2^)	0.35 ± 0.11	0.36 ± 0.10	0.71	0.089
FAZ perimeter (mm)	2.32 ± 0.36	2.64 ± 0.43	<0.001*	<0.001*
FAZ circularity index	0.81 ± 0.07	0.66 ± 0.12	<0.001*	<0.001*

OCT = optical coherence tomography; FAZ = foveal avascular zone.

*Statistical significance was found using independent samples t-test (P<0.05).

^†^Analysis of covariance.

### Association of macular VD and FAZ-related parameters

Table 3 presents the result of linear regression between predictor variables and outcome variables in a multivariate model. When all variables were considered simultaneously, age, SE, and FAZ circularity index were significantly associated with structural parameters (peripapillary RNFL thickness and macular GCIPL thickness). Deep retinal VD was significantly associated with functional parameters (MD and PSD on HVF). In addition, FAZ circularity index was positively associated with spherical equivalent (R^2^ = 0.057, P = 0.014), macular GCIPL thickness (R^2^ = 0.214, P<0.001), and peripapillary RNFL thickness (R^2^ = 0.198, P<0.001) by univariate regression analysis.

**Table 3:** Significance of independent variables in predicting outcome of glaucoma: Multivariate models.

Predictor Variables	Outcome Variables			
	Peripapillary RNFL thickness	Macular GCIPL thickness	MD	PSD
Age	0.024*	0.035*	-	0.05*
Spherical equivalent	<0.001*	<0.001*	-	<0.001*
Superfical VD	-	-	-	-
Deep VD	-	-	0.008*	0.046*
Whole retinal VD	-	-	-	-
FAZ perimeter	-	-	-	-
FAZ circulariy	0.007*	0.009*	-	-

RNFL = retinal nerve fiber layer; GCIPL = gangion cell and inner plexiform layer; MD = mean deviation; PSD = pattern standard deviation; VD = vascular density; FAZ = foveal avascular zone

* P<0.05; -, not applicable (P>0.05)

### Diagnostic ability of macular VD and FAZ-related parameters

**Fig 3:**
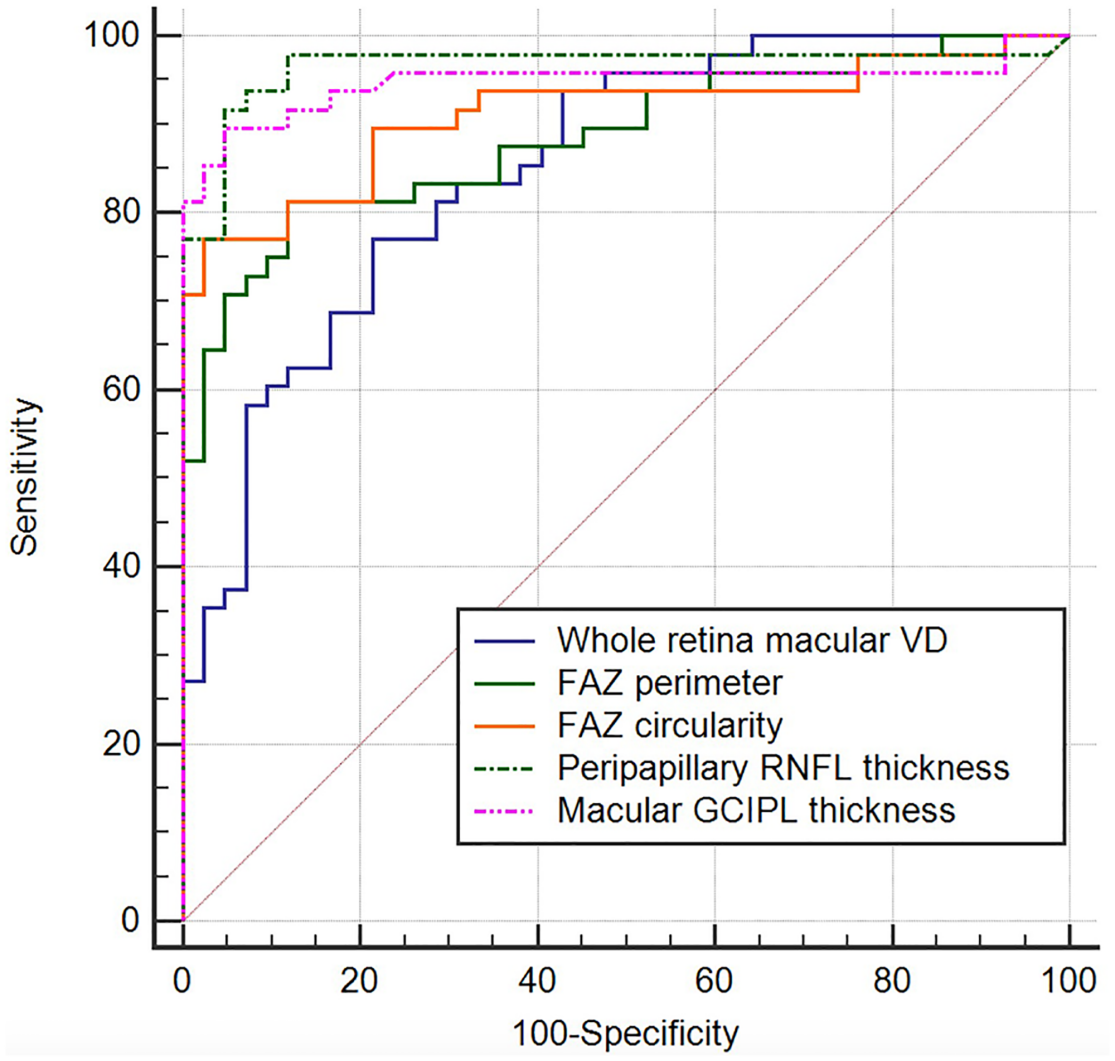
Refractive error-adjusted receiver operating curves for whole retina macular VD, FAZ perimeter, FAZ circularity index, peripapillary RNFL thickness, and macular GCIPL thickness.

Table 4 shows discriminating ability of macular VD, FAZ-related parameters, peripapillary RNFL thickness, and macular GCIPL thickness in our study group. Refractive error adjustment generally enhanced AUROC values for all parameters. Of all the macular VD parameters and FAZ-related parameters tested, refractive error-unadjusted AUROC value ranged from 0.567 to 0.866, and refractive error-adjusted AUROC value ranged from 0.793 to 0.905. The AUROC was highest for FAZ circularity index (0.905; 95% CI, 0.844–0.966), followed by temporal deep retinal VD (0.870; 95% CI, 0.803–0.937) and FAZ perimeter (0.858; 95% CI, 0.784–0.932) with refractive error adjusted. When the refractive error-adjusted AUROCs of whole retina macular VD, FAZ perimeter, FAZ circularity index, peripapillary RNFL thickness, and macular GCIPL thickness were compared, all differences remained statistically insignificant after Bonferroni correction except for the difference between the whole retina macular VD and peripapillary RNFL thickness. (Fig 3 and Table 5).

**Table 4:** Area under the receiver operating characteristic curves of macular VD and FAZ-related parameters with and without refractive error adjustment.

**Macular vascular density**		
	Refractive error-unadjusted AUROC (95% CI)	Refractive error-adjusted AUROC (95% CI)
Quadrant segmentation		
Temporal *superficial*	0.633 (0.516–0.749)	0.810 (0.730–0.891)
*deep*	0.804 (0.713–0.894)	0.870 (0.803–0.937)
*whole retina*	0.703 (0.594–0.812)	0.838 (0.764–0.913)
Superior *superficial*	0.579 (0.459–0.700)	0.794 (0.708–0.879)
*deep*	0.662 (0.549–0.776)	0.801 (0.717–0.885)
*whole retina*	0.615 (0.497–0.733)	0.802 (0.717–0.858)
Inferior *superficial*	0.674 (0.561–0.787)	0.808 (0.725–0.892)
*deep*	0.711 (0.603–0.819)	0.835 (0.758–0.913)
*whole retina*	0.708 (0.599–0.817)	0.823 (0.742–0.904)
Nasal *superficial*	0.578 (0.459–0.698)	0.809 (0.727–0.891)
*deep*	0.644 (0.530–0.758)	0.811 (0.730–0.892)
*whole retina*	0.608 (0.491–0.726)	0.813 (0.731–0.894)
Circular segmentation		
C1 *superficial*	0.567 (0.447–0.687)	0.793 (0.707–0.878)
*deep*	0.678 (0.567–0.790)	0.806 (0.723–0.888)
*whole retina*	0.607 (0.488–0.725)	0.799 (0.715–0.883)
C2 *superficial*	0.623 (0.506–0.740)	0.809 (0.727–0.890)
*deep*	0.745 (0.643–0.847)	0.840 (0.766–0.915)
whole retina	0.680 (0.568–0.791)	0.828 (0.751–0.905)
C3 *superficial*	0.664 (0.552–0.777)	0.829 (0.751–0.907)
*deep*	0.707 (0.600–0.814)	0.836 (0.760–0.912)
whole retina	0.697 (0.588–0.807)	0.842 (0.767–0.916)
Average	AUROC (95% CI)	
*superficial*	0.620 (0.503–0.737)	0.811 (0.730–0.893)
*deep*	0.726 (0.621–0.831)	0.833 (0.757–0.909)
*whole retina*	0.673 (0.560–0.785)	0.825 (0.747–0.902)
**FAZ-related parameters**		
	Refractive error-unadjusted AUROC (95% CI)	Refractive error-adjusted AUROC (95% CI)
FAZ size	0.518 (0.407–0.630)	0.781 (0.694–0.868)
FAZ perimeter	0.709 (0.610–0.808)	0.858 (0.784–0.932)
FAZ circularity index	0.866 (0.796–0.935)	0.905 (0.844–0.966)
**Reference parameters**		
	Refractive error-unadjusted AUROC (95% CI)	Refractive error-adjusted AUROC (95% CI)
Peripapillary RNFL thickness	0.961 (0.916–1.000)	0.969 (0.929–1.000)
Macular GCIPL thickness	0.942 (0.886–0.998)	0.948 (0.893–1.000)

AUROC = Area Under Reciver Operating Characteristic; CI = confidence interval’ FAZ = foveal avascular zone, RNFL = retinal nerve fiber layer; GCIPL = ganglion cell and inner plexiform layer.

The quadrant sectors were assessed in a clockwise direction for the right eyes and in an counterclockwise direction for the left eyes.

**Table 5:** Pairwise comparisons of AUROC curves referring to the glaucoma group.

	Difference between AUROCs	SE	P-value
whole retina macular VD and FAZ perimeter	0.033	0.037	0.37
whole retina macular VD and FAZ circularity index	0.059	0.044	0.18
whole retina macular VD and peripapillary RNFL thickness	0.11	0.040	0.0047*
whole retina macular VD and macular GCIPL thickness	0.096	0.043	0.025
FAZ perimeter and FAZ circularity index	0.026	0.033	0.44
FAZ perimeter and peripapillary RNFL thickness	0.081	0.036	0.024
FAZ perimeter and macular GCIPL thickness	0.062	0.041	0.13
FAZ circularity index and peripapillary RNFL thickness	0.055	0.036	0.13
FAZ circularity index and macular GCIPL thickness	0.037	0.041	0.38
Peripapillary RNFL thickness and macular GCIPL thickness	0.018	0.020	0.36

AUROC = Area Under Receiver Operating Characteristics; SE = standard error; VD = vascular density; FAZ = foveal avascular zone; GCIPL = ganglion cell and inner plexiform layer; RNFL = retinal nerve fiber layer.

*Statistical significance was found using the method described by deLong et al. Bonferroni correction was perfomed to adjust for type I error (P<0.005).

### Reproducibility

The ICCs for intersession reliability (J.K.) of FAZ size, perimeter, and circularity index measurements were 0.96 (95% CI, 0.91–0.98), 0.89 (95% CI, 0.82–0.94), and 0.93 (95% CI, 0.86–0.97), respectively. The ICCs for intergrader reliability (J.K. and J.W.S.) of FAZ size, perimeter, and circularity index measurements were 0.93 (95% CI = 0.87–0.97), 0.88 (95% CI = 0.81–0.94), and 0.91 (95% CI = 0.82–0.96), respectively.

## Discussion

In the current study, we quantitatively demonstrated the characteristics of the macular VD and FAZ in glaucoma using OCT-A. Our findings confirmed the previously known association of decreased macular VD with the development of glaucoma,[27, 37] and also showed that FAZ-related parameters had a diagnostic value for discriminating glaucoma from healthy subjects. Notably, FAZ perimeter and circularity index with refractive error adjustment performs well statistically comparable to macular GCIPL thickness and peripapillary RNFL thickness for discriminating between healthy and glaucoma patients.

Abnormalities of the circulation of the ONH, retina, or choroid have been suggested to have a role in the etiology of glaucoma.[38–41] A histologic study of 20 eye bank eyes found that eyes with advanced glaucomatous damage after long-standing POAG exhibit morphologic changes, including decreased density of choriocapillaries and large choroidal vessels.[37] Recent advances in OCT-A technology have made it available to assess VD of specific retinal layers in a given region of interest. By subtracting two consecutive B-scans, OCT-A generates microcirculatory information of the ONH, peripapillary area, or macular area. Measuring the concentration and velocity of red blood cells (RBCs) particles allows assessment of RBC flux and flow, respectively, to assess tissue perfusion and oxygen/ nutrition exchange rates within micro-structures.[42] Tissue perfusion images acquired by OCT-A have been shown to be correlated with blood flow in vivo.[43]

The macula including parafoveal region is of great clinical importance in glaucoma. In a study in which the rates of VF change were evaluated using different VF area, the progressive decline in sensitivity in the central area, particularly the central inferior zone, has the strongest association with decline in quality of life of patients with glaucoma.[44] It has also been known that OBF plays an important role in the pathogenesis of NTG. Unstable OBF represented by circadian fluctuation of 24-hour mean ocular perfusion pressure was associated with paracentral VF defect progression.[45, 46] Anatomical evidences also support that the parafoveal region deserves attention in glaucoma. Almost 50% of retinal ganglion cells (RGCs) are distributed in the macula, and studies in primates have shown that RGC density reaches a maximum within the foveal slope, approximately 0.5 mm from the foveal pit.[47] For the vascular supply, whereas the peripapillary area has a double-layered capillary support system (RNFL and GCL) and connecting capillaries, the fovea is supplied only by the single-layered parafoveal capillary arcade.[48] Physiologically, the retina and especially the macula consume more oxygen per weight than any other tissue in the mammalian body; thus, the macula is likely susceptible to hypoxic and ischemic damage.[49] The diagnostic ability of segmented macular RNFL and GCIPL to discriminate between healthy eyes and eyes with glaucoma is high.[50]

However, the association of macular vascular structure with glaucoma has rarely been studied. In a cross-sectional study in which macular VD and retinal thickness were measured, those eyes with POAG had a lower macular VD, which was strongly associated with more severe hemimacular VF defect and thinner corresponding retinal thickness; these findings imply that a diminished macular microvasculature network is closely associated with structural and functional glaucomatous damage.[51] Our study results showed that superficial macular VD was greater than the deep macular VD in all analyzed regions. This is in accordance with the histologic study that found that eyes from deceased donors have a denser superficial plexus capillary network than the deep plexus.[52]

In a previous study, Rao et al. evaluated AUROCs of the regional VD in ONH, peripapillary, and macular regions using OCT-A in eyes with POAG, and they concluded that the diagnostic ability of the regional macular VD parameters measured by OCT-A was only moderate.[27] Specifically, they showed that macular VD had significantly lower AUROC value in POAG than did the peripapillary VD. Macular VDs analyzed in their study were only of the superficial vascular plexus present in the inner layers of the retina, and the comparisons were performed without refractive error adjustment. In contrast, refractive error-adjusted analysis were performed for macular VD and FAZ-related parameters in our study. For diagnostic accuracy, our study showed that AUROC values of regional macular VD ranged from 0.793 (C1 superficial retina) to 0.870 (temporal deep retina) in regional analysis. A recent study revealed that the density of retinal capillary microvasculature is reduced in greater myopia,[53] and thus this finding necessitates the refractive error correction is needed when analyzing VD in glaucoma. The relatively higher AUROC values of macular VD reported in our study when compared to the previous one by Rao et al. may be due to the proper adjustment of refractive error.

Apart from VD, FAZ morphology has been known to be related with many pathologic conditions of the eyeball. he size and regularity of FAZ have been shown to be of both diagnostic and prognostic value in retinal diseases such as diabetic retinopathy and retinal vein occlusion.[54–57] It is also known that the disintegrity of the vascular arcades not only enlarges the maximum FAZ diameter but also alters the shape of FAZ in diabetic retinopathy.[58] In glaucoma, microcirculatory alterations in the perifovea are spatially correlated with central VF loss. Loss of FAZ circularity was significantly associated with presence of central VF defect, whereas FAZ area was significantly associated with the severity of central VF defect.[59] With the assumption that focal loss of the parafoveal capillary network alters the shape of FAZ in glaucoma, we decided to analyze the role of FAZ perimeter and circularity index as these parameters may reflect macular vascular perfusion status.

An unexpected finding in the current study was that FAZ circularity index and perimeter were among the best discriminating parameters between eyes with glaucoma and healthy controls with AUROC of 0.905 (0.844–0.966) and of 0.858 (0.784–0.932), which were statistically comparable to those of peripapillary RNFL thickness and macular GCIPL thickness. There are a few possible hypotheses for these results. First, FAZ circularity index may be the more sensitive indicator for detecting early vascular damage, as focal loss of parafoveal capillary arcade tends to happen prior to the enlargement of FAZ in advanced disease (Fig 2C–2F). Second, the relatively low diagnostic accuracy of macular VD outside foveal region may implicate that the focal loss of capillaries in the early stage of glaucoma is not well reflected in the parameter of sectoral macular VD outside fovea calculated from OCT-A images, as there is a multilayer vascular support system in both the RNFL layer and the GCL in these regions. In contrast, the subtle change of parafoveal vascular arcade may render the outline of FAZ more irregular, resulting in the decreased FAZ circularity index in early stage of the disease, as there is only a single layer of vascular support in this area.

To the best of our knowledge, there have been no available data to date on the relationship between FAZ circularity index and myopia. In a prospective cohort study of 117 healthy eyes, Tan and colleagues measured 3 x 3-mm macular OCT-A images and evaluated the impact of demographics and ocular factors, and they found that both superficial and deep FAZ size had significant correlations with axial length and SE by univariate regression analysis.[60] To minimize the confounding effect of myopia on the FAZ-related parameters, we used an analysis of covariance and the covariate-adjusted ROC curve models as described earlier in this study. In our study, FAZ circularity index was smaller in more myopic eyes. A possible hypothesis on the positive association of FAZ circularity index with spherical equivalent may be that axial length elongation in myopia has affected the alignment of the parafoveal capillary arcade.

The consistent associations of FAZ circularity index with peripapillary RNFL thickness and macular GCIPL thickness by multivariated regression analysis may imply that disrupted parafoveal vascular blood supply is related to the structural glaucomatous damage and has a diagnostic value in differentiating glaucoma from healthy eyes. Future studies are needed to investigate if our findings are applicable to the different types and stages of glaucoma.

There may be some advantages of using FAZ circularity index over conventional methods. First, as the breakdown of parafoveal capillary network is quantified, vascular risk for POAG pathogenesis may be assessed in a more exquisite way. Future research may advance the diagnosis of glaucoma if this parameter is properly combined with structural damage of GCIPL or RNFL. Second, there is no need of special devices for calculating FAZ circularity index. It can be calculated easily with OCT-A images using open source image processing program such as ImageJ.

Our study is not without limitations. First, this is not a prospective study, and normal and glaucomatous eyes had a relatively small sample size of 52 eyes for each group. To overcome this limitation, we designed a thorough age- and sex-matched case–control study. Small number of patients in each group was due to matching of the glaucomatous eyes with normal eyes by age and sex to avoid their possible effects on the measurements of FAZ metrics. This patient matching may also limit the generalizability of our findings when applied to the clinical practice. Second, there may be unknown confounding variables that can affect microvascular perfusion status of the macula such as systemic antihypertensive medications that the glaucoma patients had been receiving. Third, measurements of FAZ size, perimeter, and circularity index were based on subjective measurements using imageJ software program by our graders. However, this limitation was addressed by excellent intersession and intergrader repeatabilities exhibited by our graders (ICCs: 0.88–0.96). Development of the reliable automated procedure for assessing FAZ morphology may enhance its diagnostic value.

## Conclusions

The current study quantitatively evaluated macular VD and FAZ-related parameters using OCT-A in glaucoma. There were decreased macular VD, increased FAZ perimeter, and decreased FAZ circularity index in eyes with OAG compared with healthy eyes. With refractive error adjusted, these parameters showed considerable diagnostic value for glaucoma. In particular, FAZ circularity index showed a considerable diagnostic accuracy for discriminating glaucoma from healthy subjects. This unique finding of our study presents the possibility that FAZ circularity index may act as a novel biomarker for glaucoma diagnosis that reflects the microvascular perfusion status of the macula. In addition, we believe that our algorithm to quantitatively assess the macular capillary networks using macular VD and FAZ morphology may be of help for future glaucoma research especially in the vascular pathogenesis.

## Supporting information

S1 File Dataset of patient demographics, OCT measurements of structural parameters, and OCT-A measurements of macular vessel densities and foveal avascular zone-related parameters. (XLSX)
